# Non-Surgical Management of Apical Fenestration Associated with Apical Periodontitis in a Tooth with an Open Apex: A Case Report

**DOI:** 10.3390/reports8020076

**Published:** 2025-05-22

**Authors:** Alexander Bonchev

**Affiliations:** Department of Conservative Dentistry, Faculty of Dental Medicine, Medical University, 1431 Sofia, Bulgaria; a.bonchev@fdm.mu-sofia.bg

**Keywords:** apical bone fenestration, apical plug, immature root, immediate pre-endodontic dentin sealing, case report

## Abstract

**Background and Clinical significance**: Apical fenestration is a rarely reported clinical finding that may be associated with apical periodontitis. However, its diagnosis can often be complicated by overlapping clinical and radiographic features. While management traditionally involves a combination of endodontic and surgical interventions, there is limited documentation regarding successful outcomes achieved through non-surgical treatment alone. Therefore, further reporting and investigation of such cases are warranted to enhance clinical understanding and inform decision-making. **Case Presentation**: This case report describes the non-surgical management of a 20-year-old patient presenting with symptomatic apical periodontitis and a labial apical fenestration in a previously treated maxillary left central incisor (tooth #21) exhibiting an open apex. Diagnosis was confirmed using cone-beam computed tomography (CBCT), which revealed a bone defect in the facial cortical plate. The treatment protocol involved conservative canal debridement, intracanal placement of calcium hydroxide, and final obturation using an apical plug of calcium silicate-based hydraulic cement (CSBHC) and the monoblock technique. Over a follow-up period of two years and eight months, clinical and radiographic assessments demonstrated resolution of symptoms, healing of the sinus tract, and complete regeneration of the buccal cortical bone. **Conclusions**: This case highlights the potential for complete healing of apical fenestration associated with apical periodontitis in an open apex tooth through non-surgical endodontic treatment alone.

## 1. Introduction and Clinical Significance

Chronic apical periodontitis is a common endodontic pathology that develops as a result of pulpal disease, most often caused by complications from dental caries or trauma. It leads to pathological changes in the periapical tissues, including alveolar bone loss [1]. If left untreated, this bone resorption can progress and extend to the cortical surface of the alveolar bone, causing further complications [2]. Among the potential complications of this progression is the formation of an apical fenestration [3,4]. Apical fenestration may occur as a physiological irregularity of the bone or as result of a pathological process [5]. The American Association of Endodontists (AAE) defines apical fenestration as a window-like defect in the alveolar cortical plate that frequently exposes a portion of the root, typically on the facial aspect of the alveolar process, while the integrity of the alveolar bone margin is preserved [6]. When the marginal bone is affected, the condition is defined as dehiscence [3]. The fenestration may be asymptomatic or present with different non-specific symptoms, such as tenderness to palpation and discomfort during mastication or with potential mucosal involvement [7], known as mucosal fenestration. Due to the diverse clinical presentations of these lesions, the proper diagnosis might be difficult, and a cone-beam computed tomography (CBCT) investigation might be necessary for accurate assessment [8].

Apical fenestrations are rarely documented in the literature, and as a result, there are no clear guidelines for their diagnosis and management. Most of the published case reports describe a combined treatment approach involving non-surgical endodontic treatment followed by surgical intervention [5]. Among dental clinicians, there is a common informal belief that when fenestration affects the buccal cortical bone, surgical treatment is always indicated, often without distinguishing between the different etiologies of the condition [5].

The aim of the present case report is to demonstrate a two-year and eight-month follow-up on the management of an apical fenestration associated with apical periodontitis in a tooth with an open apex with only a non-surgical approach.

## 2. Case Presentation

### 2.1. Patient Information

A 20-year-old male patient with no known systemic diseases or medical conditions was referred to the endodontic clinic for evaluation and treatment of tooth #21. The patient reported a history of dental phobia and had experienced anxiety episodes during previous dental procedures.

The chief complaints included pain during mastication involving the maxillary left central incisor, as well as recurrent swelling in the labial vestibular region of the upper jaw. According to the patient, the tooth had undergone endodontic treatment in the past, although the specific timing and details of the procedure were unknown. No history of dental trauma was reported.

### 2.2. Clinical Findings and Diagnosis

Intraoral examination revealed localized swelling in the vestibular apical region of tooth #21, along with a suppurating sinus tract located above the projection of the root apex. Mild tenderness was noted on percussion and palpation. Periodontal probing depths were within normal limits, indicating no periodontal involvement. Pulp sensibility testing with both electric and thermal stimuli produced negative responses for tooth #21. A composite restoration was present on the tooth, with clinical evidence of secondary caries.

**Figure 1:**
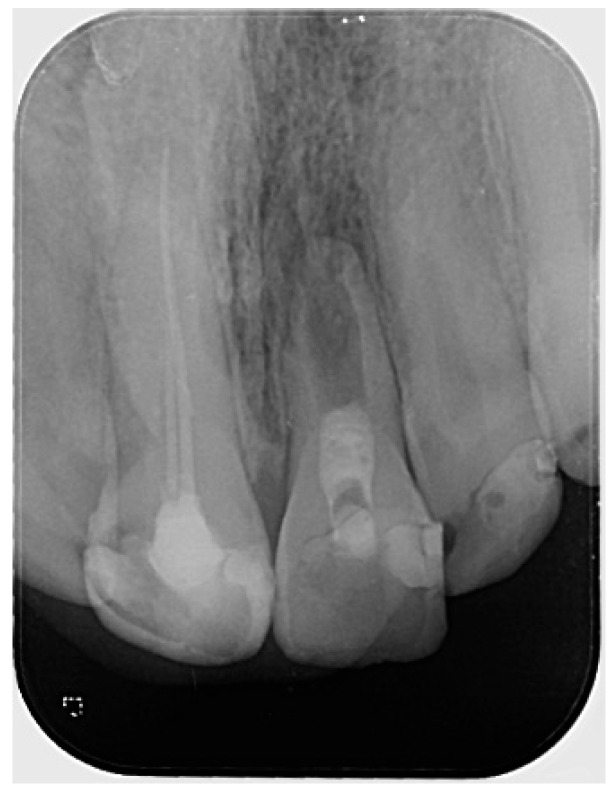
Preoperative radiograph of tooth #21. The radiograph shows previous endodontic treatment, with the root canal filling material not extending to the full length of the canal. A diffuse radiolucency is visible around the open apex. (The mark in the lower left corner indicates the left side).

The periapical radiograph showed that tooth #21 had undergone previous endodontic treatment. A diffuse periapical radiolucency was observed surrounding the open apex. Additionally, the root appeared relatively short and exhibited thinner dentinal walls compared to the adjacent tooth #11 (Figure 1).

After the examination, tooth #21 was diagnosed with symptomatic apical periodontitis. The treatment plan was discussed with the patient, presenting three possible options. The first option was non-surgical secondary orthograde endodontic treatment, which was recommended by the operator as the most conservative approach. The second option involved apical microsurgery following non-surgical treatment. The final option was extraction with subsequent implant placement.

### 2.3. Therapeutic Intervention

The patient provided written informed consent and opted for the first treatment option, non-surgical therapy. Local anesthesia was administered with 1.8 mL of articaine (40 mg) containing 0.01 mg of epinephrine, and rubber dam isolation was placed. All procedures were performed under magnification using a dental operating microscope. The existing composite restoration was removed, and any carious tissue was thoroughly excavated. An endodontic access cavity was then prepared, followed by immediate pre-endodontic dentin sealing and buildup using Optibond FL (Kerr Corporation, Orange, CA, USA) and Tetric Evoflow (Ivoclar, Schaan, Liechtenstein). The previous root canal filling material was removed with an ultrasonic tip under water cooling. Bleeding was observed from the root canal during this step. After determining the preliminary working length, the root canal walls were gently instrumented using hand K-files. Irrigation was performed with 5.25% sodium hypochlorite, which was activated using a negative pressure irrigation system (EndoVac, Kerr Corporation, Orange, CA, USA).

**Figure 2:**
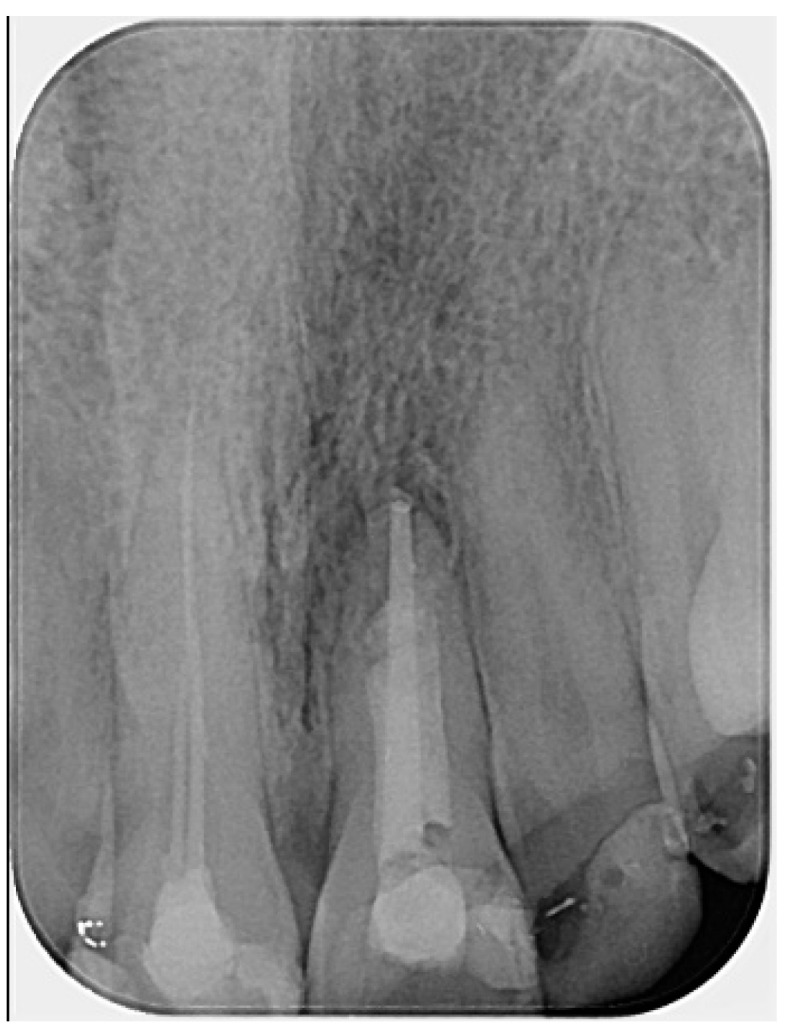
Radiographic verification of the working length confirmed accurate measurement during the clinical appointment. (The mark in the lower left corner indicates the left side.)

The working length was confirmed radiographically using an ISO size 80 gutta-percha cone (DiaDent, Cheongju, South Korea). Following this, the gutta-percha was removed, and calcium hydroxide dressing (Calcipast, Cerkamed, Stalowa Wola, Poland) was placed in the canal and left in situ for a period of two weeks (Figure 2).

At the second visit, the sinus tract persisted, and a serous exudate was observed from the root canal. Given the complexity of the case, a cone-beam computed tomography (CBCT) examination was performed for further assessment.

**Figure 3:**
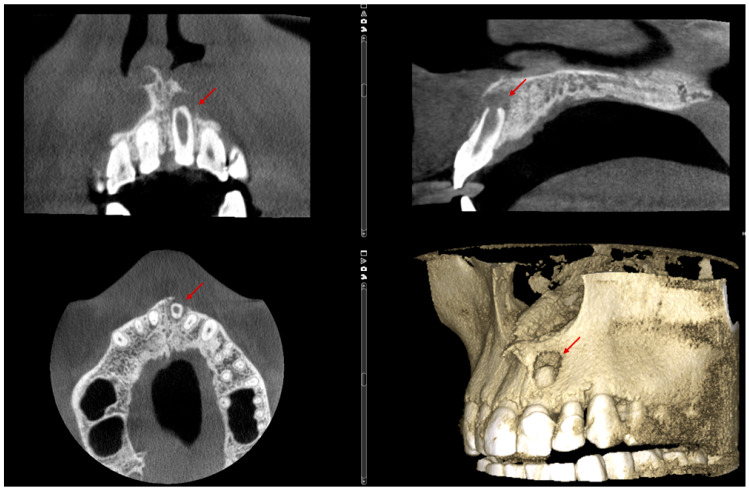
The labial bone fenestration is visible on the CBCT image (indicated by red arrows).

The CBCT scan revealed a labial cortical bone defect associated with tooth #21, measuring approximately 7 mm in diameter. The apical third of the root was uncovered by bone; an apical bone fenestration was present. The palatal cortical bone remained intact and was not affected by the periapical pathology (Figure 3). According to the CBCT Periapical Index (CBCTPAI), the lesion was classified as score 4 + D, indicating extensive periapical radiolucency with destruction of the cortical bone [9].

After the analysis of the CBCT, during the second visit of the patient, copious irrigation with activated 5.25% sodium hypochlorite was performed, followed by a second application of intracanal calcium hydroxide dressing and temporary filling of the cavity with glass–ionomer cement. The dressing remained in the root canal for an extended period of six months, as the patient unexpectedly traveled abroad and was unable to complete the treatment during that time.

At the subsequent visit, the canal was dry, the sinus tract had resolved, and healthy gingival mucosa was observed around the root apex, allowing for final obturation to be completed. Prior to root canal filling, the canal was irrigated with 17% EDTA followed by 5.25% sodium hypochlorite, activated using a negative pressure irrigation system.

**Figure 4:**
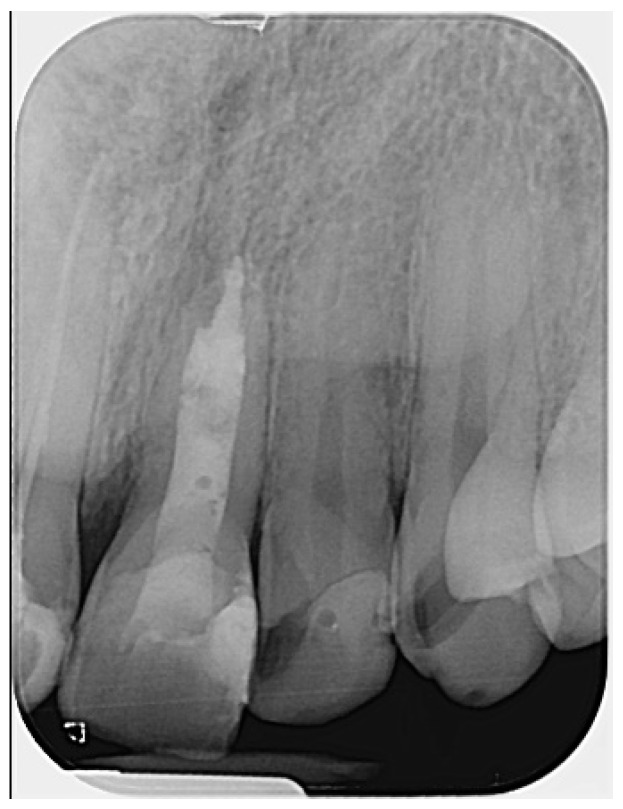
Radiograph following root canal obturation with CSBHC. Evidence of bone healing around the apex is visible, likely initiated during the period of calcium hydroxide intracanal dressing. (The mark in the lower left corner indicates the left side).

An apical plug was created using calcium silicate-based hydraulic cement (Well-Root PT™ Vericom, Chuncheon-si, Republic of Korea). Prior to placement, the plugger (Buchanan hand pluggers, Kerr Corporation, Orange, CA, USA) was adjusted to be 2 mm short of the working length. A portion of the CSBHC was delivered into the canal, condensed with a plugger, ultrasonically compacted, and then further adapted using a large paper point. After placement, a control radiograph was taken to assess the adaptation of the CSBHC in the apical third. A 4–5 mm apical plug was successfully formed. The rest of the root canal was filled with calcium silicate-based hydraulic cement, which was also condensed with the plugger and ultrasonically compacted, following the concept of primary monoblock (Figure 4).

The coronal restoration was completed with a fiber composite (EverX posterior, GC, Tokyo, Japan) in combination with a universal composite resin material (Tetric Prime, Ivoclar, Schaan, Liechtenstein).

### 2.4. Follow-Up and Outcomes

**Figure 5:**
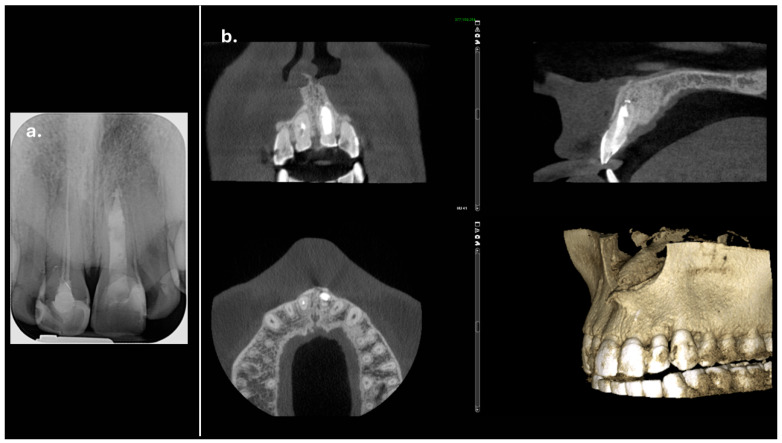

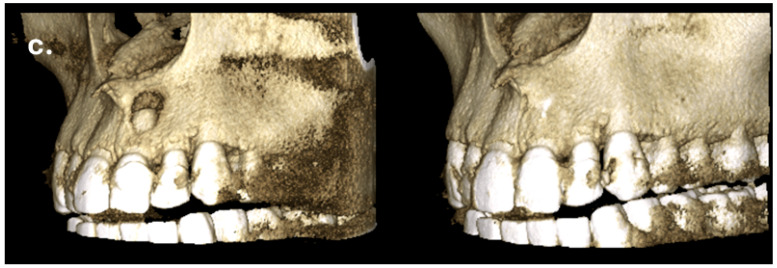
Complete regeneration of the apical bone fenestration observed over a period of two years and eight months: (**a**) periapical radiograph showing resolution of the periapical radiolucency; (**b**) CBCT scan confirming re-establishment of the cortical bone and healthy periapical tissues; (**c**) the 3D reconstruction is included to illustrate the anatomical changes before and after treatment.

The patient returned for a follow-up visit two years later. The tooth was asymptomatic, and the radiograph showed no signs of periapical radiolucency (Figure 5a). The caries lesion on the mesial surface was treated. Eight months later, a new CBCT scan was performed by another dentist due to a traumatic injury on the right side of the jaw. Upon reviewing the scan, valuable diagnostic information became apparent, including complete restoration of the cortical bone, reestablishment of healthy periapical tissues, and the absence of any radiolucency around the apex of tooth #21. The CBCT PAI score was recorded as 0, indicating complete healing (Figure 5b,c).

## 3. Discussion

Apical periodontitis is among the most frequently encountered and technically demanding conditions in endodontic practice. However, its potential association with alveolar bone defects, such as fenestrations and dehiscences, remains underreported in the scientific literature [5,10]. This may be due, in part, to the fact that fenestrations commonly occur on the buccal surface of the alveolar bone, where they are not easily detectable on standard periapical radiographs. Therefore, cone-beam computed tomography (CBCT) plays a critical role in accurately diagnosing these defects, guiding treatment planning, and evaluating treatment outcomes [11].

According to the body of scientific evidence, it is important to clearly differentiate between two types of fenestrations associated with endodontic disease. Some fenestrations are pre-existing prior to endodontic treatment, due to anatomical variations or as a result of malocclusion, trauma, or orthodontic treatment. These fenestrations are usually asymptomatic and do not require specific treatment [5,12,13]. However, in the presence of endodontic infection, a previously existing fenestration may sometimes complicate the treatment outcome. There are documented clinical cases linking persistent symptoms such as pain and discomfort following endodontic therapy to the presence of a fenestration. This may be due to the extruded filling materials that come into direct contact with the periosteum and mucosa, or mechanical irritation during digital palpation by the patient [14,15]. Treatment in such cases typically involves periapical surgery to eliminate mechanical irritants affecting the mucosa and to reshape the root in a favorable manner, thereby improving the conditions for a successful outcome.

On the other hand, an inflammatory process of endodontic origin may itself contribute to the development of a fenestration by inducing resorption of the cortical bone plate. At present, there is limited scientific evidence regarding the prevalence of apical fenestrations directly resulting from apical periodontitis [5]. It is plausible that such lesions have been observed and reported in clinical cases but were not explicitly identified or classified as fenestrations. Furthermore, distinguishing whether a fenestration is pre-existing or a consequence of chronic inflammation often poses a diagnostic challenge [16]. There are currently no established guidelines or standardized protocols specifically tailored to the management of these cases.

In the described clinical case, the periapical inflammation led to the formation of a sinus tract. However, there was no exposure of the root in the oral cavity, indicating a bone fenestration without involvement of the overlying mucosa (mucosal fenestration). This finding supported the decision to initially pursue a non-surgical approach and postpone the periapical surgical intervention in case of an unsuccessful conservative treatment outcome.

Based on the preoperative radiograph and clinical findings during the initial treatment visit, it can be assumed that the tooth may have experienced trauma during the patient’s childhood—although the patient does not recall a specific incident. Dental trauma during the stage of root formation can lead to root development arrest, which is typically characterized by an open apex and thin dentinal walls [17]. This anatomical condition presents a clinical challenge in terms of effective disinfection and sealing. The initial endodontic treatment in this case appears consistent with an attempted regenerative endodontic procedure (REP), as indicated by the radiographic presence of a calcium silicate-based material. The long-term success of REPs depends on several critical factors, including thorough disinfection, induction of intracanal bleeding, and the establishment of a hermetic coronal seal [18]. In situations where REP is unsuccessful or contraindicated—such as in older patients or following a failed prior attempt—apexification with an apical barrier using calcium silicate-based hydraulic cements offers a predictable and reliable alternative. These bioactive materials are well-documented for their ability to promote hard tissue formation, provide effective apical sealing, and support periapical healing [19]. In the present case, the decision to proceed with apical plug placement rather than a second regenerative attempt was based on the patient’s age and the presumed failure of the previous regenerative intervention.

It is generally assumed that thorough removal of microbial infection from the root canal system should lead to healing of the periapical lesion and regeneration of the cortical bone, without the need for periapical surgery [20]. According to the latest statements from the European Society of Endodontology (ESE) and AAE, in cases with an open apex and thin dentinal root walls, mechanical instrumentation should be avoided in order to prevent further weakening of the root structure. This makes the debridement protocol even more challenging to implement. Nevertheless, an alternative approach advocates gentle and minimal mechanical shaping using larger sized K- or H-files to disrupt bacterial biofilms and enhance irrigation efficiency [21]. The latter approach was followed in the management of the present case. The persistence of symptoms two weeks after the patient’s first visit may be associated with challenges in disinfecting the root canal system and the presence of a sinus tract. The application of calcium hydroxide was intended to enhance chemical disinfection, as its use is recommended in cases of severe root canal infection, particularly when a sinus tract and active exudation are present—as observed in the described clinical case. Additionally, the use of a calcium hydroxide dressing allows for the monitoring of infection control and provides the opportunity to adjust the treatment protocol if necessary [22]. In the present clinical case, the canal was filled with calcium hydroxide for an extended period of six months, not as an elective decision but as a necessity due to the patient’s limited availability for follow-up. This duration exceeds the average period reported in the literature, which typically ranges from 1 to 6 weeks [23].

Irrigation with 5.25% sodium hypochlorite, activated using negative pressure, in combination with 17% EDTA, was employed as the disinfection protocol in this case. This combination is well-supported in the literature for its effective antimicrobial activity and its ability to remove the smear layer, thereby enhancing the penetration of irrigants and medicaments into dentinal tubules [24]. When followed by prolonged intracanal medication with calcium hydroxide over a two-week period, this approach proved effective in achieving a dry, non-exudating root canal and promoting healing of the sinus tract prior to final obturation.

In this case, an apical plug with calcium silicate-based hydraulic cement was used to achieve proper sealing of the open apex. The remaining portion of the root canal was filled up to 2 mm short of the cementoenamel junction (CEJ) using a CSBHC, following the concept of a primary monoblock [25]. The premixed version of calcium silicate materials, Well-Root PT™, was used in this study due to its easier application and its proven biocompatibility, as well as physicochemical and mechanical properties comparable to those of MTA and Biodentine [26]. Additionally, Well-Root PT has been reported to exhibit good marginal and apical adaptation when used as an apical plug material. Furthermore, animal studies have shown that the extrusion of calcium silicate-based materials can lead to the formation of hydroxyapatite in the periapical tissues [27].

Prior to initiating the endodontic procedure, the coronal dentin was etched, treated with an adhesive system, and sealed with a thin layer of flowable composite resin. This technique, known as pre-endodontic immediate dentin sealing, was performed to protect the dentin and preserve its bonding capacity by minimizing the detrimental effects of sodium hypochlorite on the hybrid layer. The final restoration was completed using a short-fiber-reinforced resin composite in combination with conventional composite. This approach aimed to create an internal adhesive ferrule and reinforce the tooth structure without the need for a radicular post [28,29].

Successful treatment at the two-year follow-up was confirmed through clinical examination, absence of patient complaints, and a periapical radiograph. Despite the diagnostic advantages of CBCT, it is not recommended as a routine imaging method for follow-up, even in limited field-of-view settings, in order to minimize patient radiation exposure [14,30]. However, eight months later, the patient experienced a traumatic injury to the right frontal facial region, and a CBCT scan was performed by another clinician. Under these circumstances, it became possible to observe and confirm three-dimensional healing of the lesion, as well as reconstruction of the buccal cortical bone plate, indicating closure of the fenestration. These findings, also visible on the periapical radiograph, support the effectiveness of the non-surgical endodontic treatment approach in cases of bone fenestration.

Although almost three years after treatment the patient remains asymptomatic and the radiographic findings indicate a favorable outcome, there were several factors that influenced the treatment procedures. In this case, the patient was thoroughly informed about the importance of regular follow-up visits and the recommended schedule. As noted in the literature, endodontic maintenance protocols generally advise closer monitoring in the first year following treatment—typically every 3 to 6 months—and then extend to once every 6 to 12 months, depending on clinical and radiographic findings [31]. However, despite repeated encouragement and education, the patient demonstrated poor compliance with follow-up recommendations. This was largely due to pronounced dental phobia and anxiety, including episodes of panic attacks that occurred during treatment sessions. The patient declined pharmacological support to manage these anxiety-related responses, which necessitated shorter, more focused visits and ultimately limited the number of clinical appointments that could be scheduled and completed. These psychological and behavioral factors, though beyond the clinician’s control, are unfortunately not uncommon in real-world practice and can complicate the course and outcome of endodontic treatment [23]. Although the inclusion of clinical photographs would have added visual clarity to the presentation, patient-related factors prevented documentation, which must be acknowledged as a limitation.

The radiographs also revealed some imperfections, such as slight inhomogeneity in the root canal filling and a small amount of radiopaque material extruded into the periapical area. This was first observed in the gutta-percha cone trial radiograph (Figure 2), where calcium hydroxide was visible beyond the apex. This is likely attributed to the presence of barium sulfate (BaSO_4_), a radiopacifier commonly incorporated into commercial calcium hydroxide pastes, which is known to resist resorption when displaced into periapical tissues [32]. Such extrusion can occasionally occur, particularly in cases involving large or chronic periapical lesions, or in teeth with open apices where the natural apical constriction is compromised. The radiopaque material remained visible in subsequent radiographs (Figure 2). It is also possible that the material observed in the later images corresponds to a small extrusion of the calcium silicate-based hydraulic cement used for the apical plug. The presence of this material did not result in any clinical symptoms or adverse effects, nor did it appear to interfere with the periapical healing process, as confirmed by radiographic follow-up. While this finding may be considered as a technical imperfection, it did not compromise the overall favorable outcome of the present case. Nevertheless, such issues should be avoided whenever possible. Although the treatment followed in this case was adapted to patient-specific constraints, and therefore may slightly deviate from standard endodontic protocols, it provides a clinical perspective on the potential for non-surgical healing in cases of apical fenestration.

## 4. Conclusions

In this clinical case, a non-surgical treatment approach proved effective in managing apical bone fenestration associated with apical periodontitis in a tooth with an open apex. Favorable clinical and radiographic outcomes were observed after a follow-up period of two years and eight months, avoiding the need for a more invasive and costly surgical intervention. Nevertheless, extended follow-up periods and additional case reports are necessary to draw more definitive clinical conclusions regarding the long-term efficacy of this conservative approach.

## Data Availability

The original contributions presented in this study are included in the article. Further inquiries can be directed to the corresponding author.

## Abbreviations

The following abbreviations are used in this manuscript:

CBCT	cone-beam computed tomography
CBCTPAI	cone-beam computed tomography periapical index
ESE	European Society of Endodontology
AAE	American Association of Endodontists
CSBHC	calcium silicate-based hydraulic cement
